# ABS/Silicon Dioxide Micro Particulate Composite from 3D Printing Polymeric Waste

**DOI:** 10.3390/polym14030509

**Published:** 2022-01-27

**Authors:** Noura Al-Mazrouei, Ahmed Ismail, Waleed Ahmed, Ali H. Al-Marzouqi

**Affiliations:** 1 Chemical and Petroleum Engineering Department, UAE University, Al-Ain P.O. Box 15551, United Arab Emirates; 201311706@uaeu.ac.ae (N.A.-M.); 201531560@uaeu.ac.ae (A.I.); hassana@uaeu.ac.ae (A.H.A.-M.); 2 Engineering Requirements Unit, UAE University, Al-Ain P.O. Box 15551, United Arab Emirates

**Keywords:** composite, ABS, silicon dioxide, 3D printing

## Abstract

In this paper, Acrylonitrile-Butadiene-Styrene matrix composites reinforced with Nano-silica dioxide particles were examined and prepared to study their mechanical properties. The composite sheets were pre-prepared using the hot extrusion process. Due to its wide characteristics, silica dioxide additions can strengthen the usability and mechanical features of composite thermoplastics and polymers. Furthermore, introducing silica dioxide as a filler in various attributes can help to maintain the smooth flow of sufficient powders, reduce caking, and manage viscoelasticity. Despite its advantages, 3D printing generates a significant amount of waste due to limited prints or destroyed support structures. ABS is an ideal material to use because it is a thermoplastic and amorphous polymer with outstanding thermal properties that is also applicable with the FFF (Fused Filament Fabrication) technique. The findings showed that increasing the silica dioxide content reduces the tensile strength to 22.4 MPa at 10 wt%. Toughness, ductility, and yield stress values of ABS/silica dioxide composites at 15 wt% increased, indicating that the composite material reinforced by the silica dioxide particles improved material characteristics. It is essential to consider the impact of recycling in polymer reinforcement with fillers. Furthermore, the improved mechanical qualities of the composite material encourages successful ABS recycling from 3D printing, as well as the possibility of reusing it in a similar application.

## 1. Introduction

Plastic wastes have a negative impact on the environment, leading to significant pollution worldwide. Since the 1990s, plastic wastes have been considered a serious crisis and have been publicly reported. Contaminants such as persistent organic pollutants can be attracted to plastic wastes because many toxic compounds are hydrophobic, and plastic waste most likely acts as a sink for those contaminants, which can greatly affect the marine environment, especially if they are buried on the seabed [1,2]. Moreover, plastics are long-chain polymeric compounds and are either synthetic or semi-synthetic. They can be easily molded and manufactured, and they are cost-effective materials with outstanding all-around qualities [2,3,4]. High-density polyethylene (HDPE), low-density polyethylene (LDPE), polyvinyl chloride (PVC), polystyrene (PS), polypropylene (PP), and polyethylene terephthalate (PET) are the most widely used polymers [2,5].

Today, a diverse range of petroleum-based synthetic polymers is manufactured on a global scale of around 140 million tons per year, with significant quantities of these polymers entering the environment as industrial waste. Plastics are difficult to degrade because of unstable enzymes that are capable of degrading synthetic polymers in nature. Moreover, plastic waste is hazardous, since its pigmentation includes a large number of trace elements that are harmful to living organisms [6,7,8,9].

Plastics are used in various applications, such as bottles, food packaging, electrical items, and construction materials. One of the most important applications of plastics is packaging. Generally, packaging accounts for around 40% of all plastic materials used worldwide [10]. Unfortunately, bottles, bags, packaging materials, containers, cups, and lids are just a few of the plastic wastes found. For its non-biodegradable and resistive nature, plastic waste presents a significant environmental crisis [2,11,12].

Nowadays, plastics are considered essential elements in the booming 3D printing technology. Chemical and physical recycling of 3D printed waste plastics have been developed to decompose waste 3D printed polymers into valuable molecules, reuse them, or make them harmless. Many 3D printing materials are commercially available, including Polylactic Acid (PLA), Acrylonitrile Butadiene Styrene (ABS), Polycaprolactone (PCL), Polyamide (PA), and Polycarbonate (PC). The need to adopt innovative solutions for the rapid recovery of waste is crucial in the face of environmental problems necessitating the reduction of plastic waste, particularly materials such as single-use plastics [13,14,15].

On the other hand, many polymers are biodegradable, recyclable, and biocompatible. Non-biodegradable plastics can be replaced with biodegradable polymers to minimize environmental effects. Biopolymers are environmentally safe materials. Some of the biodegradable polymers include PLA (polylactic acid), PCL (Polycaprolactone), PGA (polyglycolide), PBSA (Polybutylene succinate), PHA (Polyhydroxyalkanoates), and PHB (Polyhydroxybutyrate). These polymers represent an ideal choice for recycling and using as eco-friendly packaging materials [16,17,18,19].

Additionally, recycling polymer wastes would benefit scientific and business interests and could become an essential part of the global economy strategy [20]. The most suitable and easily recycled polymers are thermoplastic polymers. PP (Polypropylene), LDPE (Low-Density Polyethylene), PEHD (Polyethylene high-density), PVC (Polyvinyl chloride), PMMA (Polymethyl methacrylate), PC (Polycarbonates), PS (Polystyrene), and ABS (Acrylonitrile butadiene styrene) are different types of thermoplastic polymers [21,22,23]. These types of polymers have remarkable physical, thermal, and electrical characteristics, allowing them to be used in a variety of applications. Because thermoplastic polymers are affordable, lightweight, and long-lasting, they may be molded into a wide variety of items to be used for many applications. Therefore, researchers and scientists are focusing on several techniques to recycle polymer wastes [24,25,26,27].

3D printing is commonly used around the world in the fields of agriculture, healthcare, automotive manufacturing, and aerospace manufacturing [28,29]. Fused filament fabrication (FFF) is a commercially available type of additive manufacturing technology; it allows for new possibilities for the manufacture of complicated geometries in a variety of materials. FFF is designed and used for modeling and prototyping to build elements that are affordable in cost and simple to operate. It could be used in the aerospace, automobile, and medicinal industries [30]. In the FFF procedure, the thermoplastic filament is fed into a heated nozzle, melted, and then extruded and placed in the desired shape mold, resulting in the formation of the desired 3D object. The FFF technique has shown incredible benefits, including less waste, minimal cost, and simplicity of material modification [31].

3D printing produces a substantial amount of waste from defective prints or discarded support structures despite its benefits.

Polymer recycling and the use of agro-industrial wastes are examples of efforts that contribute to the development of a sustainable society. As a result, several studies have indicated that the mechanical properties of recycled polymers are enhanced by using additives as reinforcement [32,33,34]. 

Similarly, emerging technologies such as 3D printing enable present production processes to be replaced with more sustainable ones. Traditional manufacturing techniques might continue to waste useful supplies. Instead of creating an object from a single piece of plastic, 3D printing allows for layer-by-layer manufacturing. Furthermore, because of its versatility in processing materials and customization possibilities, 3D printing adds to sustainable design. Fused Filament Fabrication constructs materials by layering 3D model data on top of each other. Complex forms may be created with this 3D printing method, which reduces material waste and production time [35,36,37].

That said, with the increasing use of 3D printing with a wide range of benefits, it is becoming more essential to develop recyclable, cost-effective, and environmentally friendly printing technologies and printable materials [22,38]. Selective material separation, decontamination and purification, grinding, re-melting, and extrusion are all part of the polymeric material recycling process for 3D printing. The hot-melt extrusion process can also be used to recycle thermoplastic printed parts and thermoplastic waste from industry and everyday life [39,40,41].

There is a wide range of polymeric materials used in FFF, such as PLA, nylon, PC, and ABS [42]. In order to obtain the intended outcome, a proper material and suitable technology should be used. A popular bio-based polymer, polylactic acid (PLA), is currently employed in a vast range of applications as a matrix material in conjunction with a variety of nanoparticle fillers. Fillers such as micro-scale fiber, nano-, or micro-scaled additives have been recommended for PLA composites in medical products, construction, and many other applications requiring advanced mechanical and thermal characteristics [43]. On the other hand, ABS is an excellent material to be used, as it is a thermoplastic and amorphous polymer that has superior thermal qualities. In addition, high roughness, dimensional stability, processability, and chemical resistance are all features of this material. ABS has been the most popular engineering polymer due to these benefits combined with a low price. ABS is well-suited to injection molding and is thus used in a wide range of manufactured items, such as automobile components, sports equipment, and toys like Lego blocks [44,45,46].

Acrylonitrile-butadiene-styrene copolymer has been adopted by many researchers to recycle polymers or a host polymer for a variety of reasons, including the fact that it is widely used and adapted for FFF 3D printing, which has mechanical properties that are significantly favorable. On the other hand, ABS is well-known for its exceptional stiffness and robustness and represents a significant portion of current plastic waste. These plastics are already being actively studied as commercial plastics for 3D printing and account for a significant portion of the recycled plastics derived from automotive waste [47,48,49,50].

Arunprasath et al. published a study about the dynamic mechanical performance of ABS, showing that it has excellent mechanical, electrical, and thermal properties [51]. Furthermore, Savvakis et al. examined ABS properties, which demonstrated a tensile strength of 22 MPa and a tensile elastic modulus of 1627 MPa. ABS shows superior mechanical performance, chemical resistance, and good processing characteristics; therefore, it is considered an engineering thermoplastic in the engineering industry [52].

Polymeric materials in their natural state have drawbacks, such as low strength and hardness and low operating temperatures, which severely restrict their application. Thus, filler additions were introduced by many researchers, showing an improvement in the mechanical properties of the polymer and achieving better outcomes. Furthermore, the primary goal is to increase the material’s thermophysical, electrical, and magnetic properties, as well as to reduce friction wear and flammability. Initially, the primary purpose of polymer filling was to lower the material’s cost by adding a low-cost filler. Lately, one of the most diversified types of materials is reinforced polymer composites, which are being used for applications in a variety of science and technology sectors [53,54,55,56].

Silica dioxide with the formula of SiO_2_ is one of the notable filler additions used in numerous experiments. SiO_2_ has been classified as a biologically safe material that may be consumed in the agriculture and pharmaceutical industries with no known health risks, despite being crystalline. In general, silica provides features that could be mixed with many polymeric materials to increase ultimate characteristics and qualities. In addition, silica has been extensively used as a filler to improve the mechanical behavior of polymeric materials [57,58]. The preparation of SiO_2_ nanoparticles is relatively easy and cost-effective. In addition, SiO_2_ can be found in a variety of fields. It is commonly applied in catalysts, chemical sensors, chromatography, and ceramics [59,60].

According to the published literature, the addition of silica as reinforcement increases polymer composite qualities such as elastic modulus and tensile strength [61,62,63]. Experimental research conducted by Rahmani and Rostamiyan shows the behavior of ABS-based nanocomposites reinforced with nano-silica dioxide particles. The paper claims that nano-silica dioxide particles are used to strengthen ABS-based nanocomposites. The static and dynamic strength of the manufactured nanocomposites were significantly improved by increasing the silica dioxide content up to 2%. The manufactured samples, however, became brittle as the silica dioxide level was increased further [64]. In addition, an investigation addressing the process for manufacturing new polymer matrix composites with nanosized barium ferrite (BaFe_12_O_19_) as ferrimagnetic filler, acryl butadiene styrene (ABS) as a polymer matrix, and an extrusion-based method, fused filament fabrication (FFF), as 3D printing method was published by Hanemann et al. to study the composite material’s mechanical properties [65].

The researchers stated that the mechanical properties of ABS could be enhanced with the addition of silica dioxide filler, and that ABS is a material that can be easily injected, molded, and processed [66,67].

In the current research, ABS was mixed with sand silica dioxide at various filler concentrations to form ABS/SiO_2_ thin-film composite sheets. A series of mechanical tests were performed on the composite thin-film ABS/SiO_2_ sheets to investigate mechanical properties like tensile strength, elastic modulus, ductility, yield stress, and toughness. 

## 2. Material and Methods

Various wastes of ABS from 3D printing were collected from the university’s prototype laboratories, sorted by color, and destroyed using a shredding machine. In the first step of the shredding process, a robust DIY shredding machine (commercial machine) with stainless steel blades was used to break down the recyclable waste material from the 3D printing machine into small pieces [68,69,70,71]. Since the plastic waste originated from lab sources and was thoroughly sorted, the shredded material was subsequently broken into smaller fragments using a heavy-duty mixer equipped for hardwood (commercial machine). The shredded material did not contain shredder residue.

Silica dioxide sand was collected from a local area, cleaned with water, dried, and then sieved to speed up the grinding process. The silica dioxide sand used in the study was classified into three different types (carbonates, silicates, and free silica dioxide, mostly in the form of detrital quartz). According to the outcomes of the study, silica dioxide has a mineral composition of 36.93 wt%. The mineral composition of silica dioxide is 47.17 wt% silicates, 26 wt% carbonates, and up to 14 wt% quartz [72].

**Figure 1:**
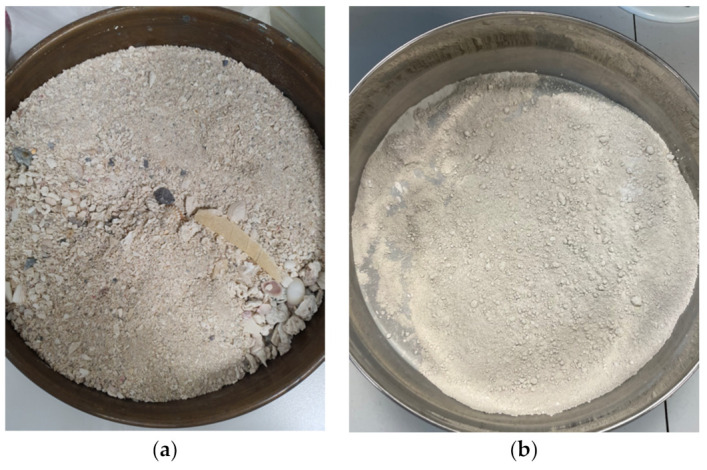
(**a**) Sand silicon dioxide sample before grinding; (**b**) sand silicon dioxide sample after pre-griding.

The sieved particles were processed with several grinding procedures using a heavy-duty grinder (Planetary PM-400, Retsch, Haan, Germany). To have a better mix with the ABS, silica dioxide was ground several times to produce a smaller particle size. Figure 1 shows a successful dry-grinding attempt that included a 10 min pre-grinding phase and a 50 min fine-grinding operation. The material was dried before grinding, to ensure that moisture did not cause caking effects.

**Figure 2:**
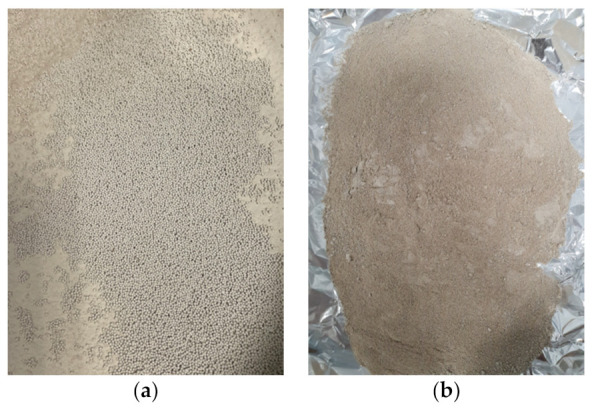
(**a**) Sand silicon dioxide sample before grinding; (**b**) sand silicon dioxide sample after pre-griding in PM 400 with 10 mm balls.

Using eight 30 mm ZrO_2_ grinding balls in a Planetary Ball Mill (PM 400, Retsch, Haan, Germany), a 250 g silica dioxide sample was pre-ground in a 500 mL Zirconia grinding jar. The powdered silica dioxide sample was caking slightly after 10 min at 320 rpm (interval reverse mode). Thus, the 30 mm balls were replaced with 150 balls of 10 mm size, as illustrated in Figure 2.

A few drops of ethanol were added to minimize the caking effects, followed by fine grinding with the 10 mm balls at a speed of 120 rpm, after 10 and 20 min. The sample was barely caking at first, but after another 30 min, the sample was visibly caking.

**Figure 3:**
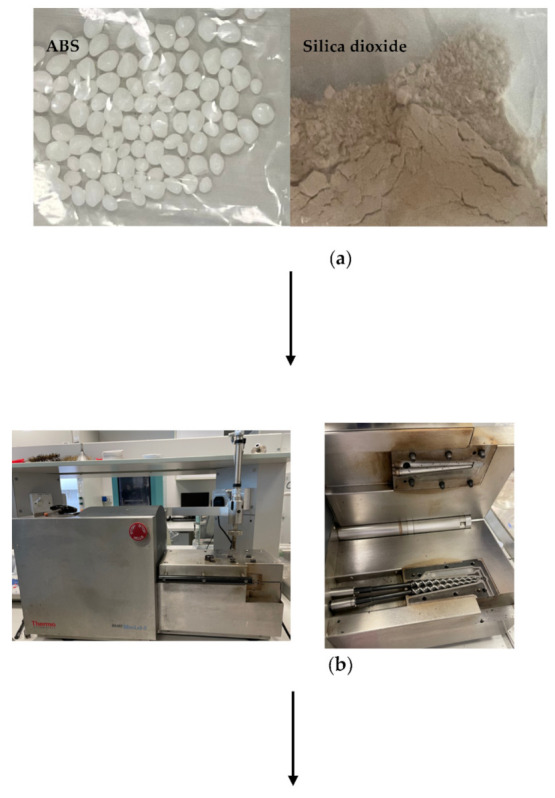

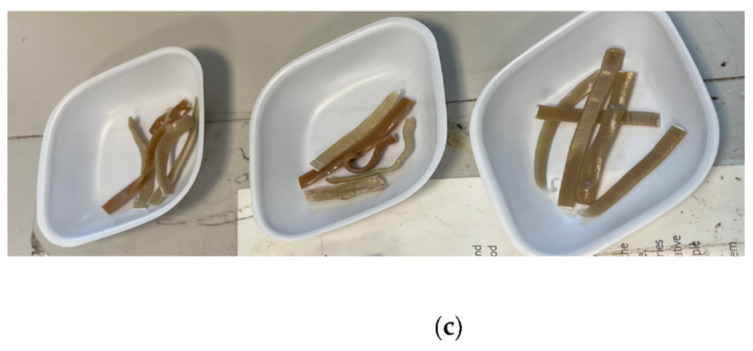
(**a**) Shredded ABS and sand silica dioxide; (**b**) twin-screw extruder; (**c**) extruder filament (ABS and silica dioxide) samples.

ABS leftover filaments were shredded into small pieces using a mechanical shredder. The extruder feed hopper had an estimated length of 10 mm; therefore, the shredded pieces were small enough to fit in the hopper. In this study, three distinct mixing ratios (5 wt%, 10 wt%, and 15 wt%) of powdered silica dioxide microparticles (~3 μm) were blended with ABS. The microparticles and ABS were mixed in the exact proportions to create composite samples, which were then produced in a melt blender with a twin-screw extruder (MiniLab HAAKE Rheomex CTW5, Karlsruhe, Germany). To stabilize the optimum mixing properties, the whole set of composites as well as a pure ABS were processed in a closed-loop cycle for 5 min at 190 °C with a screw rotational speed of 140 rpm. Furthermore, as illustrated in Figure 3, the extruder was used to blend and force the material out of the extruder through a valve to collect the mixture as filament for the next stage. The control sample was produced by processing pure ABS (0 wt% silica dioxide particles) under identical conditions as in previous composite samples [73,74,75]. The extruded composites were chopped into small pieces (1 g) and thermally compressed for 5 min using a Carver’s press (Carver^TM^ lab presses 3891, Wabash, IN, USA) under 34.47 MPa pressure and a temperature of 190 °C to produce composite sheets that were used for the mechanical investigation.

**Figure 4:**
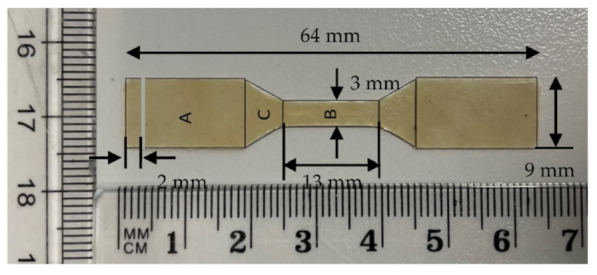
Tensile test sample dimensions.

Mechanical properties of the composite sheets were examined using the Universal Testing Machine (UTM, Shimadzu, Kyoto, Japan) to investigate the impact of the added silica dioxide microparticles on the characteristics of the generated ABS/silica dioxide composites. The final phase of the process (thermal compression) resulted in sheets, which were used to prepare samples for tensile test following ASTM D638 and ASTM D882. According to ASTM D638 and ASTM D882, test specimens for rigid and semirigid plastics in the shape of sheet, plate, and molded plastics shall conform to the dimensions described. The Type I specimen is the preferred specimen and shall be used where sufficient material having a thickness of 7 mm or less is available. The Type II specimen is recommended when a material does not break in the narrow section with the preferred Type I specimen. The Type III specimen must be used for all materials with a thickness of greater than 7 mm but not more than 14 mm. The Type IV specimen is generally used when direct comparisons are required between materials in different rigidity cases. The Type V specimen shall be used where only limited material having a thickness of 4 mm (0.16 in.) or less is available for evaluation, or where a large number of specimens are to be exposed in a limited space [76,77,78]. Figure 4 illustrates dimensions based on ASTM D638. The tensile test sample was prepared using a customized manual blanking machine (Exacta Model-JFP). 

Since the clearance between the punch and the die is represented as a ratio to the thickness of the sheet to be blanked, one of the concerns related to this procedure was that this clearance should be proportional to the thickness of the sheet. This might damage the sheared edges and generate rough surfaces, affecting the blank’s mechanical qualities and possibly leading to failure. Because the blanking machine was designed for the thin film blanking process, it is likely to have a rougher surface for thicker sheets, resulting in more significant degradation of the samples’ mechanical characteristics. The degradation of mechanical characteristics, in general, is influenced significantly by stress concentration at the edges. Additionally, tool cutting edge wear would have a significant adverse effect on sample roughness and, therefore, failure [79,80]. Furthermore, extensive pressure is applied to the blanking edge of the punch and die during the blanking process, resulting in complicated stress distribution and a variety of intersecting surface geometry and burr size [81,82].

Test specimens in the shape of a dog bone were made from composite sheets of 13 (1) × 3 (w) × 0.22 (t) mm ^3^ for 0 wt%, 5 wt%, and 10 wt% and of 13 (1) × 3 (w) × 0.26 (t) mm for 15 wt%. ASTM D 38 is the most common testing standard for determining the tensile properties of reinforced and non-reinforced plastics, whereas ASTM D882 is used for testing plastic sheets for thicknesses less than 1 mm. With the use of plastics being at an all-time high, manufacturers must be able to properly gauge the mechanical strength of their materials. Both ASTM D638 and ASTM D882 are applied through application of a tensile force to a sample specimen and measurement of various properties of the specimen under stress. In general, the tensile test was conducted on a universal testing machine at tensile rates ranging from at 1 mm/min until the specimen failed. Though ASTM D638 measures many different mechanical properties, there are different test methods for various types of plastics. ASTM D638 only applies to rigid plastic samples between 1 mm and 14 mm in thickness [83,84]. Therefore, ASTM D882 is used for testing thin plastic sheets with a thickness less than 1 mm.

**Figure 5:**
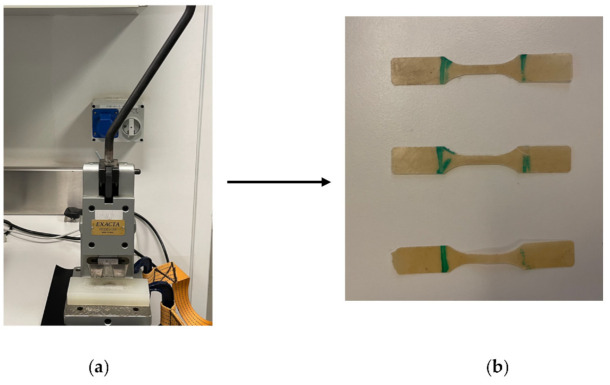
(**a**) Blanking machine; (**b**) dog bone shape samples after blanking process.

**Figure 6:**
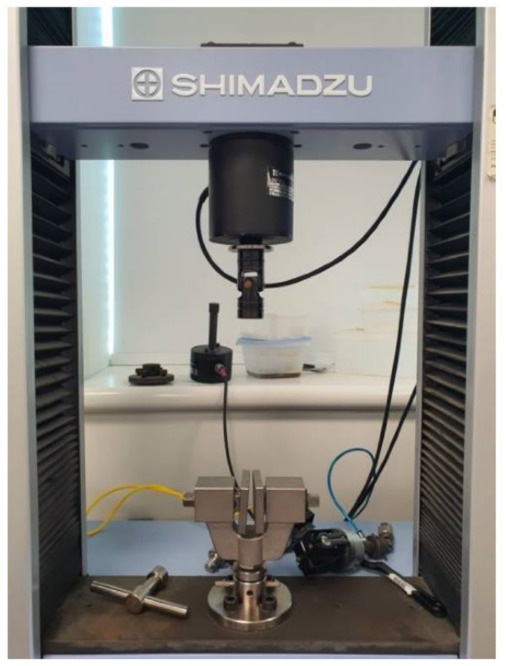
Tensile testing machine.

It is important to note that the filler content was increased from 10 to 15 wt%, resulting in increased sheet thickness. The processing is strongly linked to this variation in dimension. By allying a tensile load on a tensile machine with a 10 KN load cell, the mechanical performance of samples was evaluated. At room temperature, the linear crosshead speed was adjusted to 5 mm/min. All of the produced samples were subjected to a series of five repeated experiments. Figure 5 represents the prepared samples during the blanking process, while Figure 6 represents the tensile testing machine applied.

ASTM D638 and ASTM D882 were used to evaluate the mechanical properties of the tested samples. The tensile strength of a specimen is computed by dividing the recorded maximum load in newtons by the average original cross-sectional area of the gage length segment in square meters, as shown in Equation (1). For engineering design, elongation values are statistically significant and appropriate. Nominal strain points are determined when non-uniform deformation (such as necking) appears within the specimen gauge length [85]. Ductility is obtained by finding the prolongation (i.e., change in gage length) at the point where the specimen fractures and then dividing the estimated prolongation value by the original gage length and multiplying by 100, as illustrated in Equation (2). By prolonging the initial linear component of the load extension curve and dividing the difference in stress corresponding to any segment of the section on this straight line by the corresponding difference in strain, the modulus of elasticity can be determined as shown in Equation (3) [86,87]. Elastic modulus values were obtained by using the specimen’s average original cross-sectional area in the gage length segment. On the other hand, for samples where no proportionality is evident, the secant value is calculated by drawing a tangent and marking off the designated strain from the yield point where the tangent line goes through zero stress. Toughness was determined by calculating the area under the curve, as shown in Equation (4). In addition, arithmetic mean is computed, and the standard deviation is estimated using Equation (5).

(1) $$Tensile\;Strength\;\left(Pa\right)=\frac{Maximum\;load\;measured\;\left(N\right)}{Gauge\;length\;original\;area\;\left(m^{2}\right)}$$

(2) $$Ductility=\frac{L_{f}-L_{0}}{L_{0}}\;\times \;100.$$

where

*L_f_* = length of the specimen when it finally ruptures or breaks;

*L*_0_ = original gauge length of the specimen.

(3) $$E=\frac{\Delta \sigma \left(\varepsilon \right)}{\Delta \varepsilon }=\frac{F/A}{\Delta L/Lo\;}=\frac{F\cdot Lo}{A\cdot \Delta L\;}\;$$

where

*E* = Young’s Modulus (Modulus of elasticity);

*F* = force exerted on an object under tension;

*A* = actual cross-sectional area, which equals the size of the cross-section perpendicular to the applied force.

∆*L* = amount by which the length of the object changes;

*Lo* = original length of the object.

Toughness = area under the stress-strain (σ-ε) curve = σ × ε.(4)

(5) $$S=\sqrt{\frac{\left(\sum X^{2}-n\bar{X^{2}}\right)}{n-1}}$$

where

*S* = estimated standard deviation;

*X* = value of single reading;

*n* = number of measurements;

*X* = arithmetic means of the set of observations.

## 3. Results and Discussion

Mechanical properties were measured for composite samples made of ABS with different compositions of SiO_2_ (0, 5, 10, and 15 wt%). Wettability was also determined for these compositions by calculating the contact angle using the Tracker tensiometer made by Teclis France [88]. According to the Ultimaker company, for 3D printing machines and material filaments, ABS tensile strength is 43.6 MPa, yield stress is 39 MPa, elastic modulus is 2030 MPa, and ductility is 34% [89]. The composite blend samples were tested to find mechanical properties such as toughness, tensile strength, yield, elastic modulus, and ductility. More specifically, the samples were molded into a dog-bone cutting machine to perform multiple tensile tests. The produced thin-film’s thickness was measured using a Mitutoyo Thickness Gage (Model 547-526S) due to its high resolution (0.001 mm) and accuracy (0.0002). In this study, average measured values and the gauge length of the sample were recorded [90].

**Figure 7:**
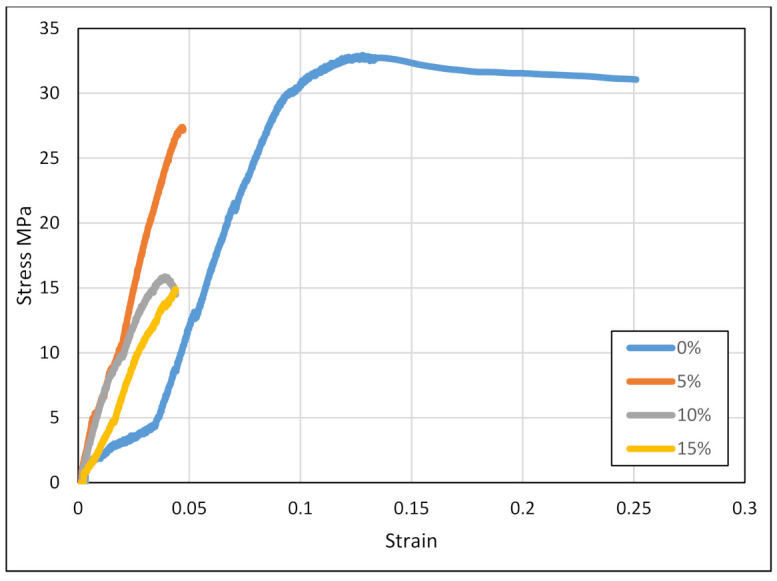
Stress vs. Strain of the composites.

As illustrated in Figure 7, the pure ABS sample has the highest breaking stress when compared to the other samples, and the value recorded was approximately 33 MPa. When 5 wt% of silica was introduced into the substrate, the breaking stress was reduced to approximately 27.5 MPa. Subsequent additions of 10 and 15 wt% silica further reduced the breaking stress to approximately 15 MPa. There was no significant difference noted between the last samples, implying that adding more silica to the ABS would have negligible effects on the stress-strain curves. Dispersion, distribution, and interaction of nanoparticles and polymer matrix determine polymer nanocomposite characteristics. A good distribution and dispersion of nanoparticles occurs when there is good adhesion in the polymer chains and the nanoparticles, and this is necessary for high breaking stress. This is the case when testing pure ABS samples. When 5 wt% silica is added to the composite, there is a decrease in the chain mobility throughout the polymer matrix volume due to the presence of the nanoparticle filler. Further additions of the silica lead to weaker mobility chains, causing more dispersion and distribution of nanoparticles. In addition, adding more silica leads to higher surface irregularities and creates voids. The result is that these defects act as nucleating agents, which leads to a decrease in the tensile strength of the composite. Some of the problems faced during this experiment include machine errors and deformation [91].

**Figure 8:**
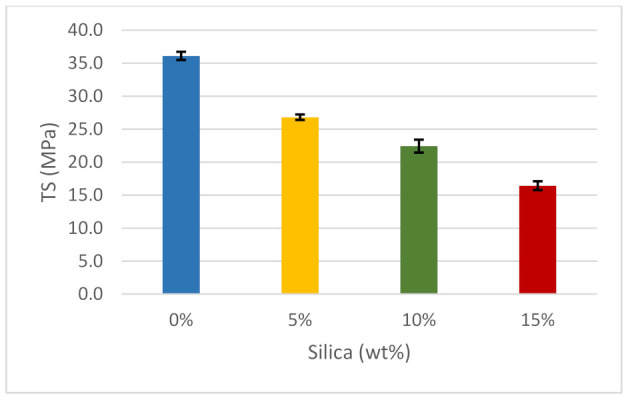
Tensile strength of the composites.

From the results plotted in Figure 8, the tensile strength of pure ABS is 36.1 MPa, which will be considered as a reference value for comparison. According to Ultimaker, ABS tensile strength is 43.6 MPa. Thus, the value obtained in Figure 8 is within an acceptable range for the pure sample. When 5 wt% of silica dioxide was added, the resulting mixture suffered from a reduction in tensile strength of approximately 25%. A further increase of 10 wt% silica dioxide caused a reduction in tensile strength to approximately 37.5%. When adding silica dioxide, the tensile strength gradually decreased, as shown at 15 wt% silica dioxide, which also led to a decrease in tensile strength to 55.6%. Generally, there is a decreasing trend as silica dioxide is added. The optimal tensile strength of the polymer was in its purest form when there was 0 wt% silica dioxide. The tensile strength reduced gradually, and it was lowest when 15 wt% silica was added to the ABS. The implication is that silica dioxide has inferior mechanical properties. Silica dioxide filler reinforcement and matrix, filler content, and filler orientation caused the recorded decrease in tensile strength. As more silica dioxide disperses in ABS, the tensile strength decreases proportionally. The agglomeration of silica dioxide particles increases when increasing the filler amount, and this significantly reduces the tensile strength. It has also been noted that void creation in samples may generate this kind of behavior in filler-matrix systems; where voids and particle clustering were identified, a significant degree of plastic deformation was discovered as well [66,92]. From the characteristics witnessed from the graphs, it can be concluded that a tensile strength involving silica dioxide only would lead to a lower maximum tensile strength.

**Figure 9:**
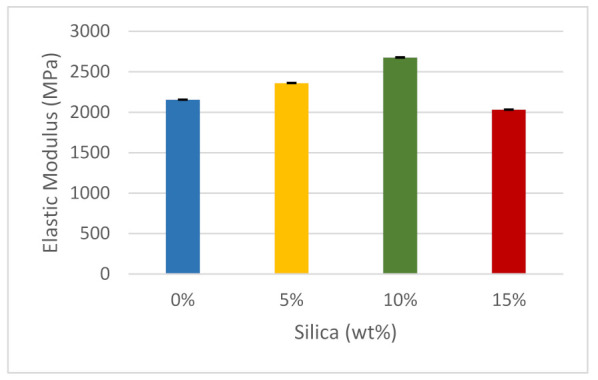
Elastic modulus of the composites.

The pure ABS elastic modulus according to the filament producer company (Ultimaker) is 2030 MPa. However, it was estimated that pure ABS had an elastic modulus of 2155.6 MPa, as shown in Figure 9. When 5 wt% silica dioxide was added, the elastic modulus increased by approximately 9.1%. When the weight percentage of silica dioxide was increased by an additional 5%, the elastic modulus increased to about 22.7%, which is the maximum value recorded. Up to this point, there was a steady increase in Young’s modulus values, but an additional 5% increase, such that the total composition of silica dioxide was 15 wt% of the total substrate, led to a 9.1% decrease in the elastic modulus. A 15% content of silica dioxide led to the lowest value of Young’s modulus, even lower than when the ABS was tested in its pure form. The general trend in the bar graph sizes indicates that the modulus of elasticity varies with the amount of strain [93]. At very low concentrations, silica dioxide seems to have remarkable properties, leading to potential improvement of ABS’s mechanical properties. As more silica dioxide is added, aggregation occurs again, which weakens the interface bonding. There is also a significant variance in the findings. This may be attributed to the fact that the dispersion of the particles is not homogeneous, since it is influenced by the mixing of the particles during the extrusion process itself [87,94].

**Figure 10:**
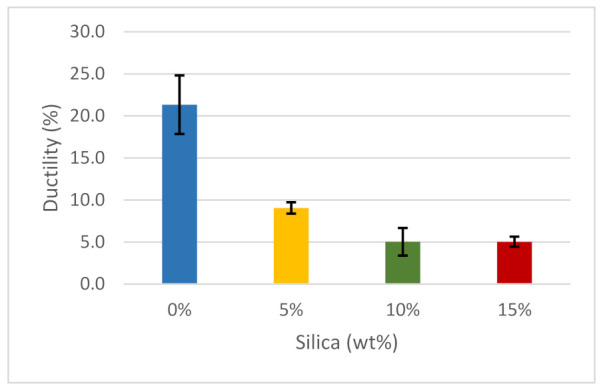
Ductility of the composites.

In general, ductility refers to the capability of the material to deform and could contribute to the energy that a sample material can sustain before reaching its breaking point. The material should adjust its plastic deformation and adapt to increasing load. Such characteristics make the material more flexible. As observed in Figure 10, a sample with 0 wt% silica dioxide had a ductility of about 21%, which was relatively higher than when samples were introduced to the silica dioxide, but it was lower than the value reported by Ultimaker (34%). There was a significant decrease in ductility when a small portion of silica dioxide (5 wt%) was introduced. As more silica dioxide was added, the ductility of the resulting material flattened to 5%. In mixing ratios 10 and 15 wt% of silica dioxide, the ductility was steady, which is attributed to the assemblage of silica dioxide particles. When increasing silica dioxide weight, clusters are formed, which leads to weaker interfaces that enable simpler interface debonding, lowering the stiffness and strength of the composite [95].

**Figure 11:**
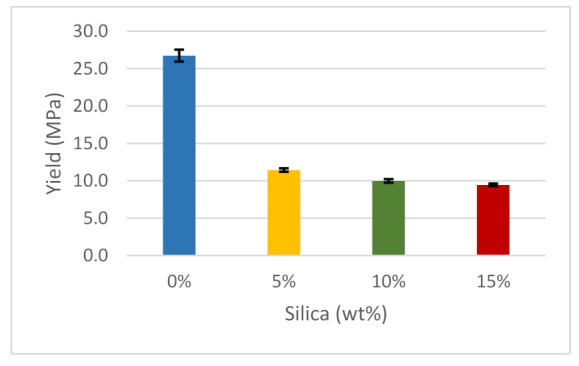
Yield stress of the composites.

Polymers generally exhibit anisotropic mechanical responses caused by variations in molecular and structural orientation. Figure 11 shows that the yield graph for the material under test follows a similar trend to the ductility graph, although there are slight differences in the results with varying concentrations of silica dioxide. A pure ABS substance had the highest yield, at approximately 26.7 MPa, whereas Ultimaker reported a yield stress of 39 MPa for pure ABS. When a small portion of silica dioxide (5 wt%) was introduced to the polymer, the yield dropped substantially, by about 55.6%. Subsequent additions of silica dioxide to the prepared material, increasing by 5%, resulted in a slight decline in the yield. The lowest yield value was recorded when the silica dioxide was 15 wt%. It is also important to note that the form of the particles has a significant impact on the uniformity of the filler. Non-homogenous mixing causes stress concentrations to arise, resulting in failures at various spots [96].

**Figure 12:**
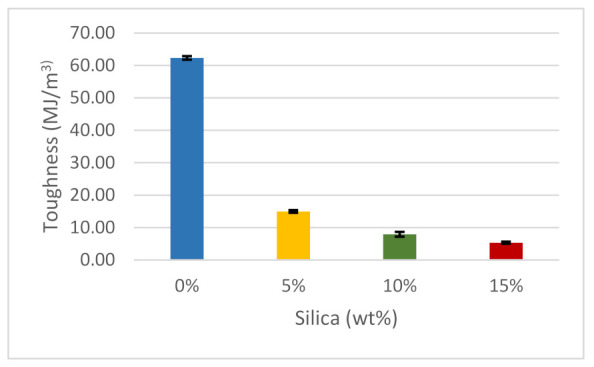
The toughness of the composites.

There is a striking similarity between the ductility and toughness graphs, and therefore it is necessary to differentiate these two properties. Toughness refers to the ability of a test material to absorb energy and undergo plastic deformation before fracturing, while ductility refers to the measurement of the plastic deformation in a material before fracturing occurs [97]. Materials can be ductile but not tough. A tough material combines high ductility and high strength. As illustrated in Figure 12, pure ABS has approximately 62 MJ/m^3^ toughness, which was the highest recorded during the toughness test. Adding 5 wt% of silica dioxide in the prepared material dramatically reduced its toughness by 75.8%, and thereafter subsequent additions of silica dioxide slightly reduced the toughness. It can be concluded that silica dioxide is not a tough material when compared to ABS. In addition, a defect may be created in a polymer composite material when it is exposed to high filler concentrations and the aggregate formation, which can lower the toughness of the material [60]. A summary of the mechanical properties of the tested samples is listed in Table 1.

According to various studies, increasing filler concentration up to 20 wt% filler addition results in a rising trend in mechanical parameters such as elastic modulus and tensile strength [97]. Because maximum mechanical characteristics were attained at 10 wt% silica dioxide addition, in this case, the addition of 15 wt% filler was used to confirm whether the trend continues to rise or decreases.

**Figure 13:**
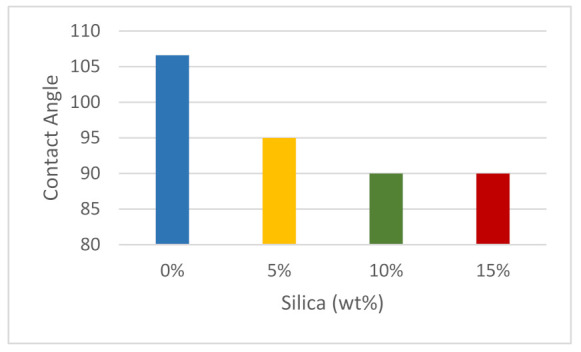
The measured contact angle of the samples.

Surface wettability is one of the most important characteristics to examine when deciding whether a material is suitable for a certain purpose, such as packaging. Wettability is calculated by using the contact angle measured on the material’s surface. To measure the contact angle, a small drop of water is placed on the surface of the sample. A contact angle of 90° or more is typical for any non-polar surface. However, a contact angle less than 90° shows that the liquid on the surface is more hydrophilic, which results in more absorption of the liquid in contact [98]. In this case, distilled water was used in a composite of different percentages of silica dioxide with ABS. As shown in Figure 13, a pure substance had the highest contact angle, around 107°, which means the wettability is low and therefore the surface is hydrophobic. When a small amount of silica dioxide was added, the contact angle decreased, indicating that the surface became less hydrophobic, which is also reported by other researchers [99,100]. When increasing the silica dioxide percentage, the contact angle decreased, whereas the wettability increased. The surface of the sample was hydrophilic in both 10 and 15 wt%, as the contact angle was 90°.

## 4. Microscopic Characterization

**Figure 14:**
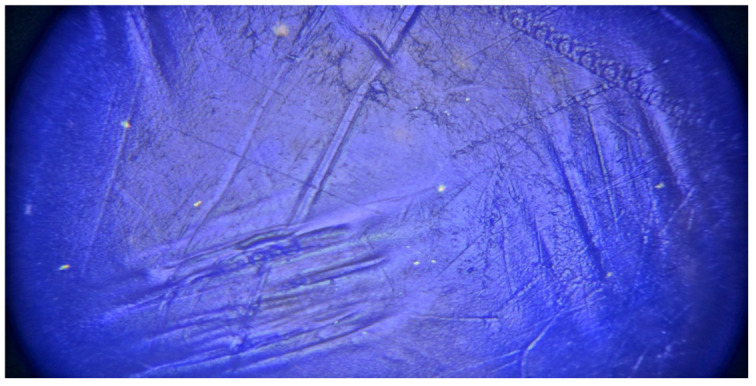
Microscope image for ABB/5 wt% silica dioxide composite.

A microscope (4000X Multi-Objective HDMI Sony IMX290 Sensor Industry Microscope, Camera Coaxial Light Monocular, Shenzhen Lapsun Technology Co., LTD, Shenzhen, China) was used to observe the surface of the neat sample of ABS and the ABS sample with 5 wt% silica. The silica nanoparticles are presented as white and spherical points, as shown in Figure 14. When comparing a neat sample of ABS and the sample with 5 wt% silica, it was observed that the neat sample had a flat surface, while the composite sample with 5 wt% silica had masses and homogenous silica in the matrix, leading to irregularity in surface and cavities. This can be attributed to the fact that silica particles are hydrophobic, and this causes lower interaction among particles. There is observable surface corrugation and voids. These defects can act as nucleation sites, and this can be used to explain the detrimental effects on the tensile behavior of the composites with increased filler loadings [91].

## 5. Thermogravimetric Analysis (TGA)

A material’s thermal resistance is defined as the highest temperature to which it may be heated without developing irreversible chemical changes that are consistent with its physicochemical characteristics. Because it allows for the study of the thermal stability of polymeric materials, including the feasibility of using materials at temperatures higher than the ambient temperature, thermogravimetric analysis is a useful tool for researchers [101].

**Figure 15:**
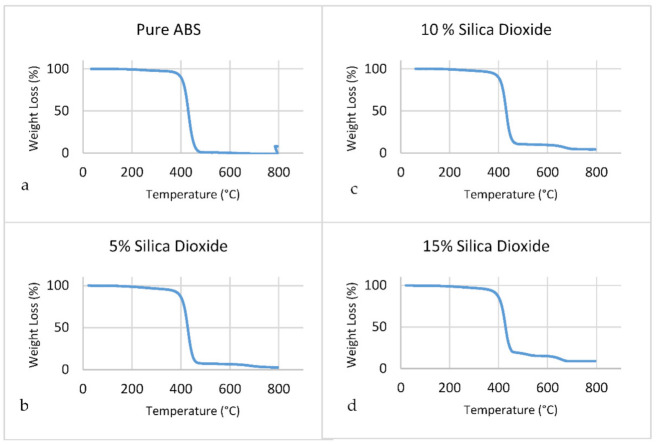
TGA results for the composites. (**a**) Pure ABS, (**b**) 5% SiO_2_, (**c**) 10% SiO_2_, (**d**) 15% SiO_2_.

As shown in Figure 15, for all samples including pure ABS, the weight loss was mostly observed between 380 to 500 °C. Because the inorganic substance does not burn away, it has better thermal stability, which is another advantage. The distribution of fillers in the polymer matrix may be responsible for the improvement in observed thermal stability [102].

## 6. Fourier-Transform Infrared Spectroscopy (FTIR)

When it comes to research, Fourier-transform infrared spectroscopy (FTIR) is one of the most essential analytical tools. Liquid, solution, paste, powder, film, fiber, and gas samples may all be classified using this method. This kind of study may also be used to examine the material on the surfaces of the substrate. The FTIR technique was created as a tool for determining organic components, including chemical bonds. During the FTIR analysis method, samples are exposed to infrared (IR) radiation for a period of time. Because of the action of the IR radiation on the atomic vibrations of molecules in the sample, a particular absorption and/or transmission of energy is seen [103,104].

**Figure 16:**
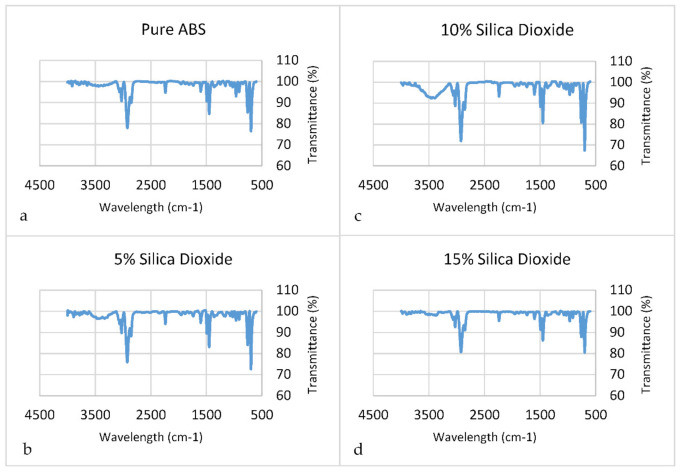
FTIR results for the composites. (**a**) Pure ABS, (**b**) 5% SiO_2_, (**c**) 10% SiO_2_, (**d**) 15% SiO_2_.

Fourier-transform infrared spectroscopy was used to examine the bonding qualities and purity of the substances under analysis. As shown in Figure 16, an FTIR test was conducted on all compositions (pure ABS and 5, 10, and 15 wt%) to study the characteristics of chemical functional groups. As shown in Table 2, it is noticeable that most wavelength values for all samples have the same functional group. Peaks of all compositions starting from 697.1 cm^−1^ have a functional group of C=C, alkene, whereas 1737.6 cm^−1^ is the C-H aromatic compound functional group. One of the strongest peaks in each figure is 2923.1, having a functional group of C-H, alkyne.

## 7. Bulk Density

The ASTM D7263–09 standard was used to determine the dry density of ground silica dioxide [105]. A controlled drying procedure in an electric vacuum oven was utilized to determine the dry density of ground sand (i.e., silica). The dry density of sand was estimated using Equation (6):

(6) $$\rho_{silica}=\frac{mass_{sand}}{Volume}$$

where $mass_{sand}$ is the determined mass of the dry silica dioxide sand, and the volume is the controlled volume of the measuring cylinder that was used.

The ABS filament’s bulk density was measured using the ASTM D792-20 standard [106]. Masses in air and water were measured and used to calculate the bulk density of ABS according to Equation (7) [106].

(7) $$\rho_{ABS}=\frac{m_{air}\;\rho_{water}}{m_{air}+m_{water}}$$

where, *m_air_* is the mass of the sample measured in air, *m_water_* is the mass of the sample measured in water, and ρ*_water_* is the density of water.

The linear mixing rule, which is a technique to estimate composite properties, built on the principle that a composite property is the volume-weighted average of the phase (matrix and dispersed phase) characteristics [107], was used to determine the theoretical density of the composite sheet, as shown in Equation (8).

(8) $$\rho_{c}=\frac{\;\rho_{filler}\;\rho_{matrix}}{\rho_{matrix\;}m_{filler}+\rho_{filler\;}(1-m_{filler})}$$

where ρ*_c_* is the density of the composite, ρ*_filler_* is the density of the silica dioxide, ρ*_matrix_* is the density of the matrix (i.e., ABS), and *m_filler_* is the mass fraction of the filler. The composite has no air accumulations, and the silica dioxide filler has no impact on the density of the polymer phase through recrystallization [107].

**Figure 17:**
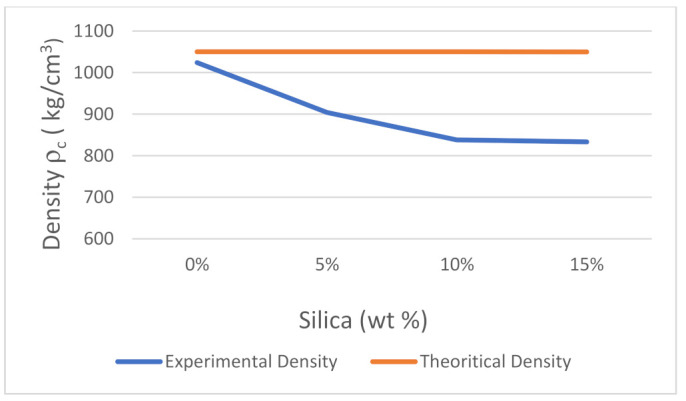
Theoretical and experimental densities.

To evaluate the experimental density of the prepared composite material, a weighing balance (Citizen-CX 220, d 0.0001 g) was used to measure the mass of the sample. To estimate the volume, the test sheets were cut using a manual cutter according to the ASTM D6287-17 standard [108]. The thickness of each composite sheet was recorded in five spots to ensure thickness consistency, using the Mitutoyo Thickness Gage (Model 547-526S). The data were used to calculate the mass-to-measured-volume ratio of the prepared samples, which was needed to approximate the experimental bulk density. The comparison of the theoretical and experimental densities of ABS/silica dioxide composite sheets is shown in Figure 17.

The calculated bulk density of the recycled ABS was approximately 1020 kg/m^3^, as illustrated in Figure 17. The experiment results show that introducing silica dioxide content led to a steady decrease in the density of the composite up to when the silica dioxide content constituted 10 wt% of the composite. The density varied from 1020 kg/m^3^ to about 840 kg/m^3^. Further increments in the silica dioxide content resulted in minor changes in the bulk density of the composite, and this can be considered as the maximum silica dioxide filler. The implication is that the silica dioxide filler has a low density when compared to ABS. A comparison of theoretical and experimental estimations shows that variations occur from approximately 0–10% of the added silica dioxide filler, and then the curves follow similar trends [107,109]. Such a trend implies that the developed composite material has thermal stability with increasing filler addition. It is a common trend for polymers to exhibit a decreasing bulk density with increased concentrations of silica dioxide particles [110]. This occurs due to the formation of air pockets during the process when high temperature is applied to the samples, thereby affecting them. Such a trend makes the composites desirable in industrial applications, since the low density means that they can easily be handled and transported [89].

## 8. Conclusions

ABS has many applications due to its advantageous properties. It is widely used in electronic enclosures in houses and factories, manufacturing companies, and house fittings [64]. In addition, according to He et al., FFF ABS components are often subjected to thermo-mechanical stresses that affect the component properties. To produce FFF ABS components with a good fatigue life, the 3D printing settings must be adjusted properly [111]. On the other hand, silica dioxide is used in medicine, the automotive industry, and water treatment facilities. Silica dioxide nanoparticles have unique characteristics, including low toxicity and being environmentally friendly, stable, and cost-effective. Introducing nanoparticles into polymers can enhance their mechanical features [112,113]. Currently, many studies are focused on producing polymer-based nanocomposites. The addition of nanoparticles can be made to any form of polymer, including thermoplastics, thermosets, elastomers, and even polymer mixtures [114,115].

In this study, the twin extruder machine was used along with compression molding to create composite sheets made of ABS and silica dioxide. In comparison to the pure ABS waste, mechanical analysis of the created material revealed a decrease in the composite material’s tensile strength values from 0 to 15%; the highest value recorded 3.61 MPa was at 0 wt% silica dioxide. Moreover, in ductility and toughness, the composite values decreased significantly with the addition of silica dioxide at both 10 and 15 wt%. The lowest value recorded was 5.4 MPa. In addition, the value recorded for toughness, 5.3%, was at 10 wt% silica dioxide. The maximum elastic modulus increased from 21.6 MPa at 0 wt% to 26.8 MPa at 10 wt%. On the other hand, there was a slight decline to 20.3 MPa after this point in elastic modulus at a silica dioxide weight of 15%. The contact features between the silica dioxide particles and the polymer are attributed to the degradation in the mechanical properties of the particulate reinformed composite [116,117]. Filling temperature, filling speed, filling sequence, film thickness, filler percentage, and manufacturing aspect are all variables that can be controlled and can affect the results significantly [118]. Aumnate et al. conducted an investigation based on producing ABS/graphene oxide (GO) composites for 3D printing applications. It was found that ABS’s tensile strength and Young’s modulus can be improved by adding GO [119].

Further research into the possibilities of employing silica dioxide combined with waste ABS for 3D printing would be beneficial. It is important to understand the impact of recycling on the reinforcement of polymer with filler and how it affects the mechanochemical stability of the final product. Furthermore, because silica dioxide is a natural material, it may be combined with biomass to improve mechanical qualities in order to create hybrid biodegradable composite goods via 3D printing [120].

## Figures and Tables

**Table 1:** The mechanical properties of the ABS/silica dioxide mixture with standard deviation (SD).

Silica (%)	Tensile Strength (MPa)	SD	Yield (MPa)	SD	Elastic Modulus (MPa)	SD	Ductility (%)	SD	Toughness (MPa)	SD
0%	36.10	±1.2	26.73	±1.6	2155.56	±3.5	21.34	±11.6	62.33	±56.2
5%	26.80	±1.0	11.44	±0.2	2361.11	±3.8	9.06	±2.7	14.98	±7.4
10%	22.43	±0.7	9.98	±0.2	2677.78	±13.0	5.04	±1.0	7.93	±3.3
15%	16.44	±0.4	9.45	±0.2	2033.33	±0.6	5.04	±1.12	5.34	±4.1

**Table 2:** FTIR results for all composites.

Pure ABS		5 wt% SiO_2_		10 wt% SiO_2_		15 wt% SiO_2_	
Wavelength (cm^−1^)	Functional Group	Wavelength (cm^−1^)	Functional Group	Wavelength (cm^−1^)	Functional Group	Wavelength (cm^−1^)	Functional Group
697.1	C=C, alkene	697.1	C=C, alkene	697.1	C=C, alkene	697.1	C=C, alkene
1449.2	C-H, alkane	1450.2	C-H, alkane	1449.2	C-H, alkane	1449.2	C-H, alkane
1737.6	C-H, aromatic compound	1954.0	C-H, aromatic compound	1737.6	C-H, aromatic compound	1733.7	C-H, aromatic compound
1952.1	C-H, aromatic compound	2237.5	C-H, aromatic compound	1952.1	C-H, aromatic compound	1953.1	C-H, aromatic compound
2237.5	C≡C, alkyne	2853.7	C≡C, alkyne	2237.5	C≡C, alkyne	2237.5	C≡C, alkyne
2854.1	C-H, alkane	2923.1	C-H, alkane	2854.1	C-H, alkane	2852.2	C-H, alkane
2923.1	C-H, alkyne	3026.7	C-H, alkyne	2923.1	C-H, alkyne	2921.6	C-H, alkyne
3027.2	C-H, alkene	3061.0	C-H, alkene	3027.2	C-H, alkene	3027.6	C-H, alkene
